# Intrauterine growth pattern and birthweight discordance in twin pregnancies: a retrospective study

**DOI:** 10.1186/1824-7288-40-43

**Published:** 2014-05-05

**Authors:** Giuseppe Puccio, Mario Giuffré, Maria Piccione, Ettore Piro, Valentina Malerba, Giovanni Corsello

**Affiliations:** 1 Dipartimento di Scienze per la Promozione della Salute e Materno Infantile, Università degli Studi di Palermo, Palermo, Italy

**Keywords:** Twins, Birthweight discordance, SGA, Weight percentile, Neonatal anemia

## Abstract

**Background:**

Twins, compared to singletons, have an increased risk of perinatal mortality and morbidity, due mainly to a higher prevalence of preterm birth and low birthweight. Intrauterine growth restriction (IUGR) is also common and can affect one or both fetuses. In some cases, however, one twin is much smaller than the other (growth discordance). Usually, high birthweight discordance is associated with increased perinatal morbidity. The aim of this study is to describe the epidemiological features of a population of twins at birth, with particular reference to the interpretation and clinical effects of birthweight discordance.

**Methods:**

We evaluated retrospectively the clinical features of 70 infants born from twin pregnancies and assessed birthweight discordance in 31 pregnancies where both twins were followed at our institution. Discordance was treated both as a continuous and a categorical variable, using a cutoff of 18%. Possible relationships between birthweight discordance and other variables, such as maternal age, gestational age, birthweight percentile, number of SGA newborns in the pair, Hematocrit (Ht) discordance and neonatal anemia, prevalence of malformations, neonatal morbidity and death, were analyzed.

**Results:**

In our cohort birthweight percentile decreased slightly with increasing gestational age. Birthweight discordance, on the contrary, increased slightly with the increase of gestational age.

A high discordance is associated to the presence of one SGA twin, with the other AGA or LGA. In our population, all 6 pregnancies in which discordance exceeded 18% belonged to this category (one SGA twin).

Ht discordance at birth is associated to the presence of neonatal anemia in a twin, but it is not significantly related to weight discordance.

Finally, in our case history, weight discordance is not associated in any way with the prevalence of malformations, morbidity and mortality.

**Conclusions:**

Birthweight discordance is an important indicator of complications that act asymmetrically on the two fetuses, affecting intrauterine growth in one of them, and usually determining the birth of a SGA infant.

Our case history shows a significant statistical association between pair discordance and IUGR in one of the twins, but we could not demonstrate any relationship between discordance and the prevalence of malformations, morbidity and mortality.

## Background

The number of twin births has doubled in the last 25 years [1], probably because of the increase in average maternal age at first pregnancy and the diffusion of assisted procreation techniques. Today more than 3% of newborns are twins [2]. Twins, as compared to singletons, have an increased risk of gestational complications, intrauterine growth restriction (IUGR), preterm birth, chromosomal abnormalities, congenital malformations and perinatal mortality and morbidity. These complications are more common in monozygotic twins [3-5].

Intrauterine growth restriction (IUGR), often manifesting itself as a SGA birth, is also common in twins, and can affect one or both fetuses. The incidence of IUGR in twin pregnancies is 25-35% [6]. Intrauterine growth of twin fetuses is generally comparable to the growth of singletons in the first and second trimester of pregnancy, while it is usually significantly reduced in the third trimester (starting approximately at 30 weeks of gestation) [7]. Increasing competition for room and resources as pregnancy goes on is a reasonable explanation for that.

Different scenarios of growth abnormalities can be observed in twins. One of the twins, or both of them, can be small for gestational age (SGA). Small differences in weight between twins can be considered physiological and related to individual genetic variations, and are not likely to affect further growth and development. In some cases, however, one twin is much smaller than the other (growth discordance). Growth discordance does not necessarily correspond to growth restriction and discordant intrauterine growth does not necessarily cause the birth of a small for gestational age (SGA) baby. However, one or both discordant twins can also be SGA and in at least 2/3 of discordant twin pairs, the smaller twin has a weight < 10th percentile. Discordant twin pairs in which the smaller twin is also SGA have an increased risk of neonatal death [8].

Usually, birthweight discordance is associated to increased perinatal morbidity and mortality [9]. Different thresholds have been proposed as cutoffs to define high discordance as a predictor of morbidity [10-17]. A recent prospective study suggested a discordance in excess of 18% as an appropriate cutoff to predict adverse perinatal outcome both in monochorionic and in dichorionic twin pregnancies [18].

In case of birthweight discordance, the smaller twin has an increased risk of morbidity (necrotizing enterocolitis, polycythemia, hypoglycemia) and mortality, while respiratory distress (RDS) seems to affect mainly the bigger twin [19]. The causes of twin growth discordance are often unknown. The most important is considered to be monochorionicity, mainly because of unequal sharing, or of velamentous cord insertions in the IUGR twin [20]. Other possible causes are “intrauterine crowding” and the presence of malformations [21-23]. Low weight gain, advanced maternal age, maternal hypertensive disorders, cigarette smoking, velamentous insertion of umbilical cord and pregnancy induction may increase the risk of fetal growth discordance in twin pregnancies [24-27].

## Methods

The aim of this study is to describe the epidemiological characteristics at birth of a case series of twins in order to better understand the meaning of weight discordance. We also considered the discordance in hematocrit (Ht) values measured at birth, and the relationship between Ht discordance and weight discordance. It is an epidemiological descriptive study: there was no specific primary outcome, and therefore no power analysis was made. This kind of study is meant mainly to recognize possible general patterns, which can be eventually analyzed in further more specific studies. In that sense, the study is not meant to answer a specific primary question.

We considered all live births from twin pregnancies whose babies were admitted to the Neonatal Unit of Azienda Ospedaliera Universitaria Policlinico “Paolo Giaccone” in Palermo, from 01/01/2010 to 31/12/2011; a total of 70 infants, whose general features are shown in Table 1. In the same time span, 6 newborns were admitted who were born from multiple pregnancies (triplets), but they were not included in this study.

**Table 1:** Distribution of infants by birthweight class according to birthweight percentile (Ines percentiles) and twin specific percentiles

	**INES percentiles**		**Twin specific percentiles**	
	**Total**	**%**	**Total**	**%**
**SGA**	20	28.57	13	18.57
**AGA**	47	65.14	53	75.71
**LGA**	3	4.29	4	5.71

We considered a total of 70 infants in 39 pregnancies. The number of infants does not correspond to the number of pregnancies because our sample included a few cases of outborn infants where one of the twins had been admitted to a different institution. Only in 31 twin pregnancies both infants were admitted to our ward. Birthweight discordance was assessed only in that subpopulation of 62 babies. All other analyses were made on the whole population of 70 infants.

33 (47.1%) of the babies were females, 37 (52.9%) males. Mean gestational age was 34.5 weeks (SD 3.8, median 36, range 24.3 – 39.6). Mean birthweight was g. 2057.5 (SD 686.1, median 2170, range 490–3710).

As ultrasound data about growth during pregnancy were not consistently available, intrauterine growth was evaluated by the weight percentile at birth. Infants were classified as SGA (below 10th weight percentile), AGA or LGA (above 90th weight percentile) using the recent INES percentiles of 2010 for the general Italian population [28].

The INES website also allows to compute a numeric percentile for each infant, through a “smoothing” method based on standard deviation. In this way, the weight percentile at birth can be treated as a numeric variable.

Infants were also categorized as SGA, AGA and LGA using specific percentiles for twins for an Australian population published by Roberts et al. [29].

In the general analysis, however, only the classification and numeric weight percentile derived from the general Italian INES percentiles will be used.

Weight discordance was expressed as the ratio between the weight difference and the weight of the biggest twin [18]:

$$\mathrm{Weight}\;\mathrm{discordance}=\left(\mathrm{heavier}\;\mathrm{twin}\;\mathrm{weight}‒\mathrm{lighter}\;\mathrm{twin}\;\mathrm{weight}\right)/\mathrm{heavier}\;\mathrm{twin}\;\mathrm{weight}$$

We categorized the 31 pairs according to the number of SGA newborns in the pair (zero, one or two), and assessed the prevalence of discordance greater than 0.18 in each group.

We then evaluated the discordance between the values of the first Ht value in the two twins, a possible indicator of Twin-Twin Transfusion Syndrome (TTTS), with the same procedure we used for weight discordance:

$$\mathrm{Ht}\;\mathrm{discordance}=\left(\mathrm{Ht}\;\mathrm{at}\;\mathrm{birth}\;\mathrm{in}\;\mathrm{the}\;\mathrm{twin}\;\mathrm{with}\;a\;\mathrm{higher}\;\mathrm{value}‒\mathrm{Ht}\;\mathrm{at}\;\mathrm{birth}\;\mathrm{in}\;\mathrm{the}\;\mathrm{twin}\;\mathrm{with}\;a\;\mathrm{lower}\;\mathrm{value}\right)/\mathrm{Ht}\;\mathrm{at}\;\mathrm{birth}\;\mathrm{in}\;\mathrm{the}\;\mathrm{twin}\;\mathrm{with}\;a\;\mathrm{higher}\;\mathrm{value}$$

All statistical analyses were done using the open source statistical software “R” [30].

## Results

Table 1 shows the distribution of infants by birthweight class according to birthweight percentile calculated using INES percentiles of 2010, and according to twin specific percentiles. As can be seen, with INES percentiles there is a very high number of SGA babies, and only a few LGAs, which is exactly what can be expected when we apply general percentiles to a population of twins. However, a slight excess of SGA babies, and a lower number of LGA, can be observed also with twin specific percentiles. perhaps because the percentiles we used were from a different population.

**Figure 1:**
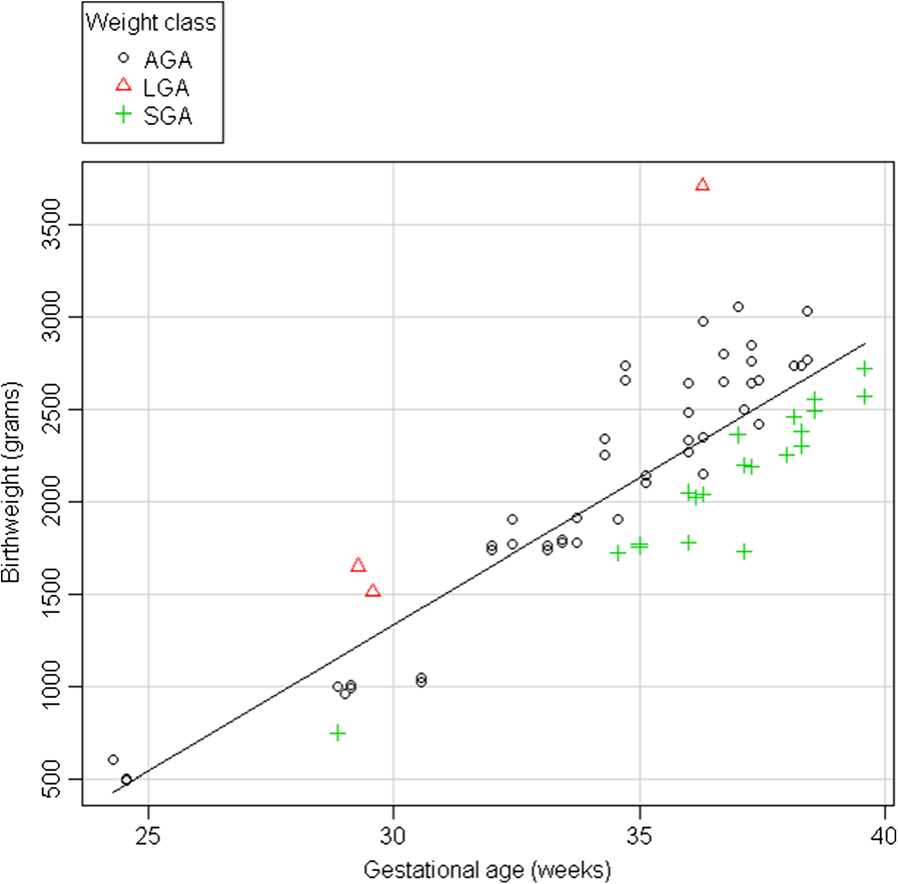
Distribution of birthweight and gestational age according to weight class calculated with Ines percentiles.

Figure 1 shows the distribution of weight and gestational age in the sample according to weight class.

**Figure 2:**
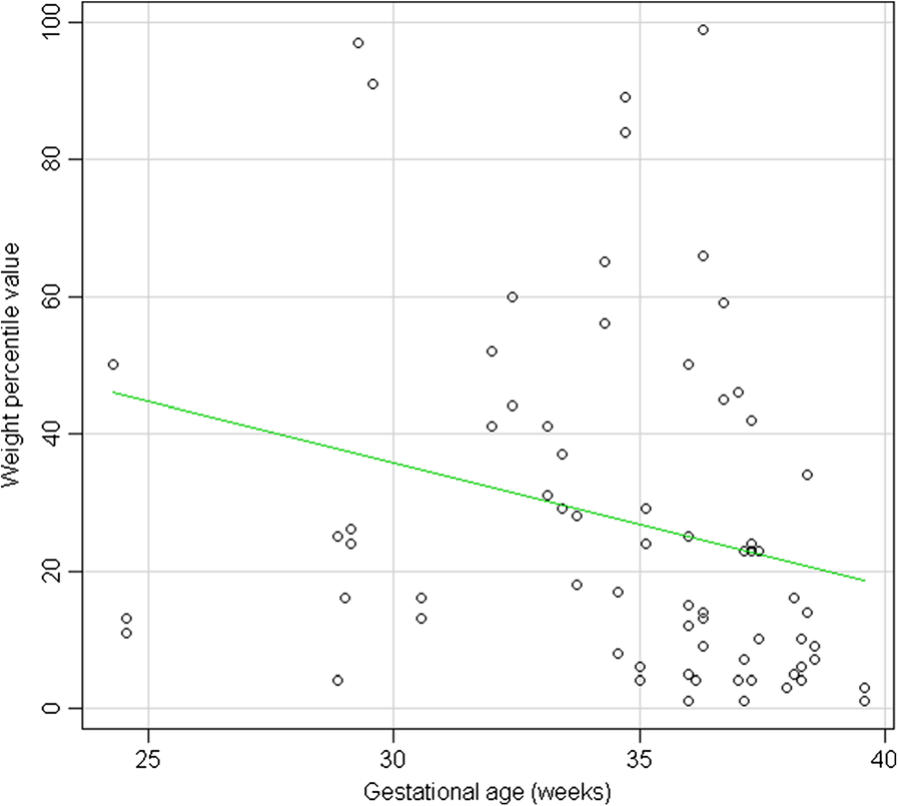
Distribution of birthweight percentiles for gestational age.

Figure 2 shows the distribution of numeric percentiles (INES percentiles) according to gestational age. Weight percentiles tend to decrease slightly, as gestational age increases. The effect, although weak, is statistically significant, as shown by a linear regression analysis: R-squared = 0.07302, p-value = 0.02368.

Information about chorionicity was available for 55/70 babies: 13 (23.6%) were born from a monochorial pregnancy, 42 (76.4%) from a dichorial regnancy. Of the 31 twin couples evaluated for discordance, information about chorionicity was available for 26 couples: 6 (23.08%) were monochorial, 20 (76.92%) dichorial.

Malformations were present in 8 cases (see Table 2).

**Table 2:** Malformations observed in our sample population

**Malformation**	**Cases**
Ventricular septal defect	1
Double renal district	1
Limb anomalies	
- bilateral phalanx hypoplasia and syndactyly of the hand	1
- adductus metatarsus	1
Cryptorchidism	2
Accessory spleen	1
Blepharophimosis	1

Important neonatal morbidity was present in 33 infants (47.14%). Only in 3 cases (4.28%) there was a neonatal death before discharge. Gestational age was significantly lower in cases with morbidity (median = 240 days versus 259 days, p = 0.0004) and death (median = 172 days versus 252, p = 0.007).

The mean value of discordance in the whole group of 31 pregnancies was 0.082, with a SD of 0.077 and a median of 0.055. Considering 0.18 as a threshold value [18], only 6 out of 31 pregnancies (19.35%) exhibited “high” discordance.

Weight discordance tended to increase slightly with the increase of maternal age, but the effect did not reach statistical significance in our limited number of cases (R ^ 2 = 0.09, p = 0.10). Weight discordance also tended to increase slightly with the increase of gestational age, but again the effect is not statistically significant (p = 0.167).

**Figure 3:**
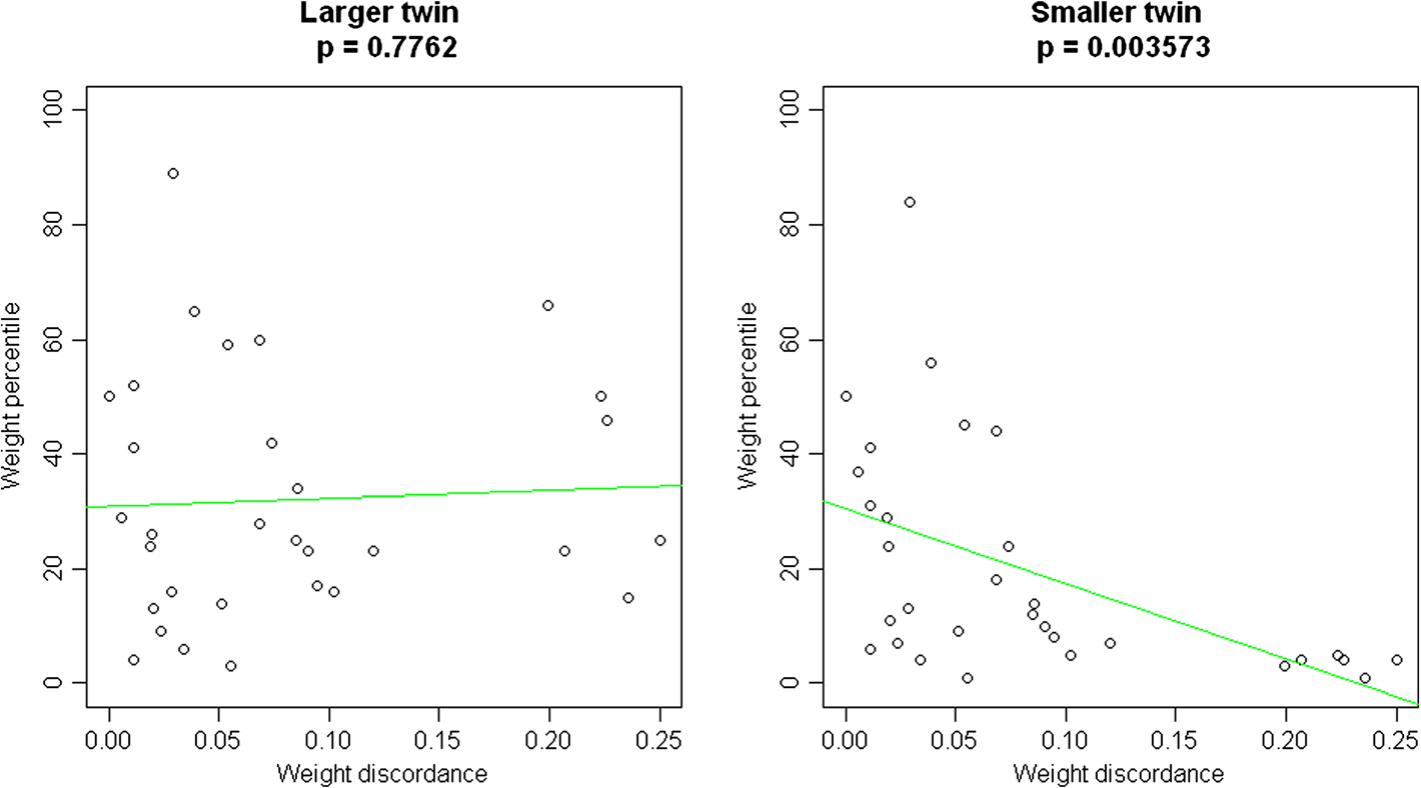
Scatterplot of birthweight percentile and weight discordance, considering separately the larger twins and the smaller twins.

There seems to be no significant relationship between birthweight discordance in the pair and weight percentile of each newborn (p = 0.10), when both twins are considered. However, if we consider separately the larger twins of each couple or the smaller twins of each couple, it is possible to see that a definite and significant relationship exists between discordance and the weight percentile of the smaller twin, but not of the larger twin (Figure 3).

Table 3 shows the distribution of weight discordance and discordance class in the 31 pairs according to the number of SGA newborns in the pair (zero, one or two). It is interesting to observe that in all cases (6 pregnancies) in which discordance exceeds 0.18, only one of the two twins is SGA. This difference is highly significant (p = 1.639e-07). On the contrary, both the 17 pregnancies in which no infant is SGA and the 4 pregnancies in which both infants are SGA, have not high discordance values. A similar result can be observed for discordance as a continuous variable: mean and median discordance values are definitely higher in the group where only one of the newborns is SGA (Kruskal Wallis test: p = 0.0004).

**Table 3:** Weight discordance and discordance class according to the number of SGA infants in each pair

**Number of SGA in the pair**	**Discordance**			**Discordance > 18%**	
	**Mean**	**SD**	**Median**	**no**	**yes**
**zero**	0,04	0,03	0,03	17	0
**one**	0,16	0,07	0,2	4	**6**
**two**	0,03	0,02	0,03	4	0

**Figure 4:**
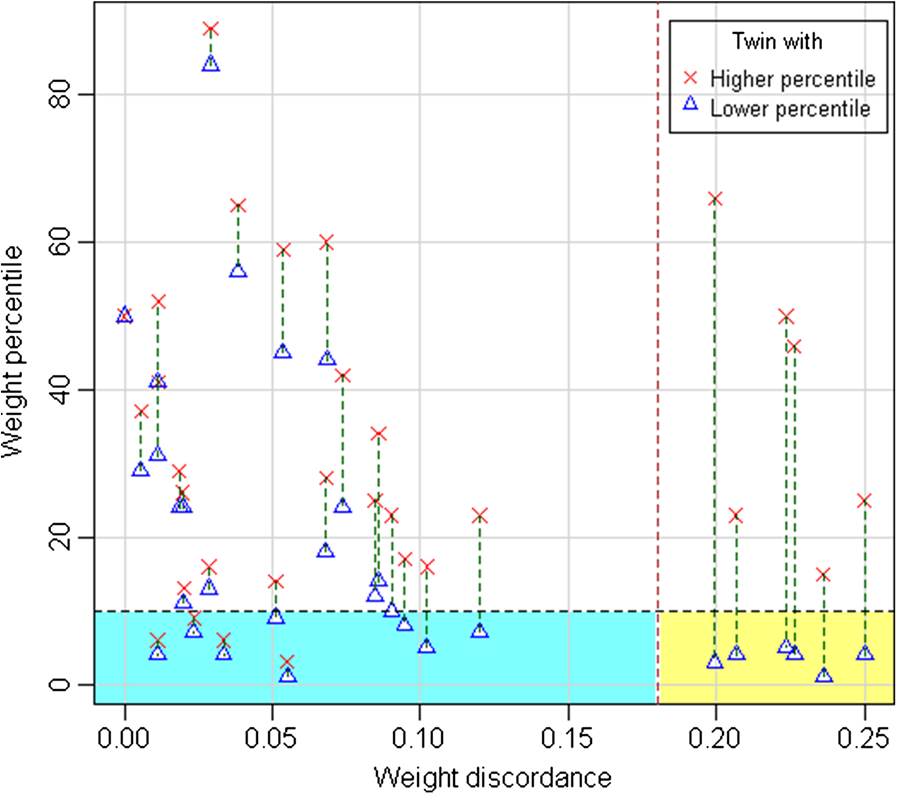
**Scatterplot of birthweight percentile and weight discordance, highlighting the behavior of individual pairs.** Blue rectangle: SGA babies in twin pairs with low discordance. Yellow rectangle: SGA babies in twin pairs with high discordance.

Figure 4 sums up these results. The blue rectangle (bottom left) includes SGA babies with low discordance, and in many of these cases both twins are SGA, especially when discordance is very low. The yellow rectangle, instead (bottom right), corresponds to SGA babies with high discordance. In these cases, the twin with higher weight percentile is always AGA.

**Figure 5:**
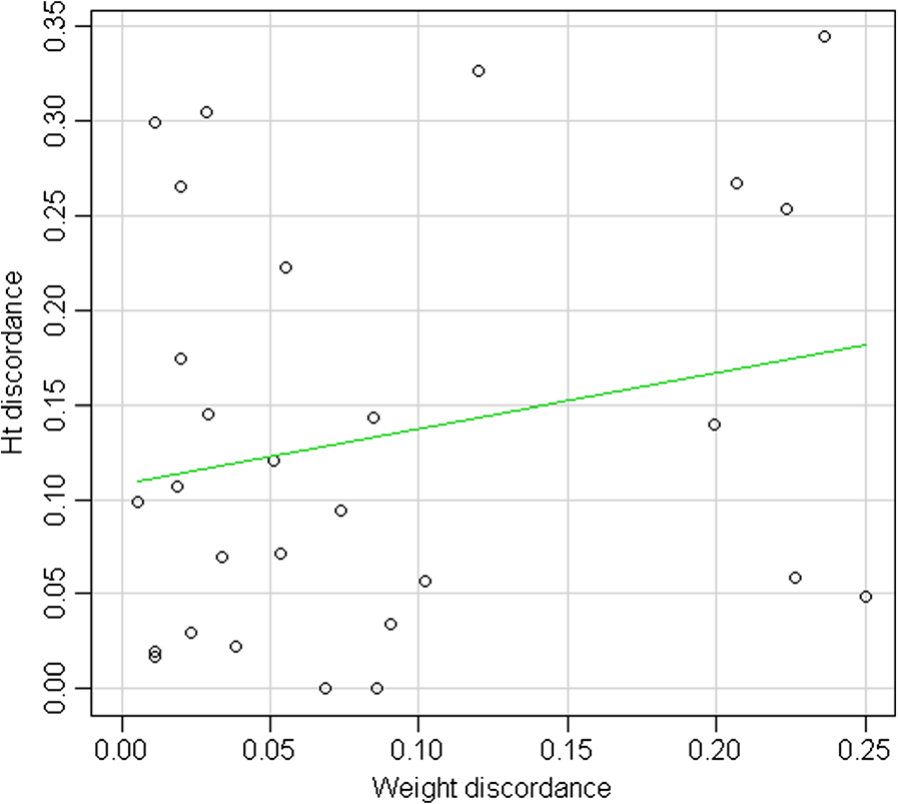
Distribution of Ht discordance according to weight discordance.

As shown in Figure 5, Ht discordance is not significantly related to weight discordance (p = 0.27).

Ht discordance is significantly higher in pregnancies in which one twin is anemic (Ht < 50%), as shown in Table 4 (Wilcoxon test: p = 2.241e-05).

**Table 4:** Mean and median of Ht discordance according to the presence of anemia

**Anemia**	**Ht discordance**	
	**Mean**	**Median**
**yes**	0,23	0,27
**no**	0,11	0,08

**Figure 6:**
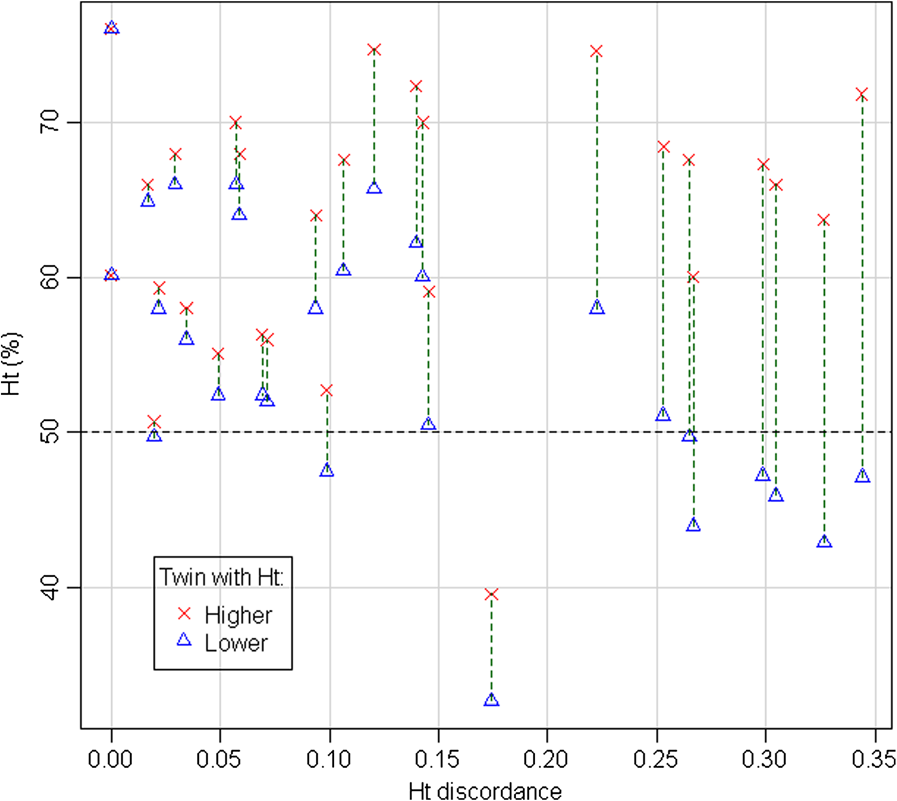
Ht values, Ht discordance and anemia.

Figure 6 shows Ht values and Ht discordance in all pregnancies. Out of 10 cases of neonatal anemia (Ht <50%), 6 are characterized by high discordance of Ht values in the pair (>0.20), with one anemic twin and the other one with high Ht values (in one case > 70%), a scenario compatible with a twin-twin transfusion. However, only two of these couples were monochorionic, while the other four were dichorionic. In two cases, discordance was low, with one of the twins just below the threshold of 50%, and the other twin slightly above. In one case both twins were anemic, with moderate Ht discordance (0.17). This was a monochorionic pregnancy with placental abruption at 24 weeks + 4 days.

Malformations were present in 8 infants (12.9%). In no cases both twins were malformed. The mean discordance was similar in the groups with and without malformations (0.079 versus 0.082, p = 0.93). Similarly, there is no significant difference in the mean discordance in the groups with and without neonatal morbidity (0.078 versus 0.087, p = 0.62).

Three children died before discharge: two were extremely preterm twins (24 + 4 weeks of gestational age) from the same monochorionic biamniotic pregnancy. Their weight discordance was very low (0.02). The third one was born from a monochorionic monoamniotic pregnancy, at 28 + 6 weeks of GA. The discordance was high (0.25). The baby who died was the one with higher birth weight. In all three cases, death was caused mainly by serious respiratory distress.

## Discussion

In our sample population we observed a very high prevalence of SGA births: 28.57% using general percentiles for the Italian population, 18.57% using specific percentiles for twins (from an Australian population). This can be explained in part if we consider that our center is a reference center for twin births, assisted reproduction, neonatal pathology, malformative and surgical pathology, and therefore a great number of high-risk pregnancies and of infants with serious neonatal pathologies are transferred to our hospital from other centers.

In our sample population weight percentile decreases slightly with increasing gestational age, probably as a result of a slight reduction of the growth rate in the last stages of pregnancy. Weight discordance, on the contrary, increases slightly with the increase of gestational age. These data are compatible with the well-known mechanical and trophic problems that affect intrauterine growth in the last weeks of a twin pregnancy.

High discordance is often associated to the presence of one SGA twin, while the other is AGA or LGA. In that case, discordance can be easily interpreted as the consequence of different growth of the two twins. Moreover, discordance seems to be related to a growth reduction in the smaller twin, and has apparently no relationship with the weight of the larger twin. In that sense, high discordance can be interpreted as a consequence of a pathological IUGR in the smaller twin, while the larger twin is not affected.

In our sample population, indeed, all six pregnancies in which discordance exceeded 0.18 belonged to the “one SGA twin” category. In pregnancies in which neither of the two twins was SGA, instead, discordance was low. Discordance was also low in all four pregnancies in which both twins were SGA. In these cases, the reduced intrauterine growth was similar in both twins.

In other words, SGA babies in twin pairs seem to belong to two different categories:

a) pairs where both twins are SGA, and discordance is low (blue rectangle in Figure 4). These cases are probably “physiological SGAs” (constitutionally and genetically smaller), or may be the results of pathological causes that affect symmetrically both twins.

b) pairs where one twin is “normal” (AGA) and the other is SGA, and discordance is high (yellow rectangle in Figure 4). In these cases, the SGA twins are probably the result of true pathological processes of IUGR, affecting only one of the twins.

High Ht discordance at birth, predictably, was often associated with the presence of neonatal anemia in one twin. In the two monochorionic couples, that could be explained as the result of a twin-twin transfusion syndrome (TTTS) or of twin anemia polycythemia sequence (TAPS), or of acute peripartum TTTS. Unfortunately, no specific diagnosis could be assigned in our two cases, due to the lack of specific obstetric or neonatal diagnostic information. Moreover, as high Ht discordance with anemia in one twin was also observed in four dichorionic couples, other mechanisms must be responsible in those cases.

In our sample Ht discordance was not related to weight discordance, perhaps because factors that determine growth restriction do not coincide with possible causes of twin-twin transfusion. Neonatal anemia was present in both twins only in one case, a monochorionic pregnancy with placental abruption. That suggests that Ht values too can be influenced by particular endouterine situations acting on both twins.

The prevalence of malformations was very high, even for a twin population (12.9%). Such a high prevalence can be explained considering that our division of neonatology is a reference center for malformations and for surgical pathology.

Finally, in our case history, weight discordance is not associated in any way with the prevalence of malformations, morbidity and mortality, but our number of cases is too small to draw conclusions on these points.

## Conclusions

Weight discordance is usually related to factors that act asymmetrically on the two fetuses, affecting the intrauterine growth of one of them, often determining the birth of one SGA infant, with all the risks associated with IUGR, while the growth of the larger twin is apparently unaffected.

Our case history confirms the significant statistical association between high discordance and IUGR, but we could not demonstrate any relationship between discordance and the prevalence of malformations, morbidity and mortality.

High Ht discordance is usually associated with anemia in one twin and can be the result of a twin-twin transfusion.

## Competing interests

The authors declare that they have no competing interests.

## Authors’ contributions

GP made substantial contributions to conception and design of the study, and to analysis and interpretation of data, was involved in drafting the manuscript and revising it. MG made substantial contributions to conception and design of the study, was involved in drafting the manuscript and revising it. MP made substantial contributions to acquisition and interpretation of data, was involved in drafting the manuscript. EP made substantial contributions to acquisition and interpretation of data, was involved in drafting the manuscript. VM made substantial contributions to the design of the study, to acquisition of data and to analysis and interpretation of data, was involved in drafting the manuscript. GC made substantial contributions to conception and design of the study, was involved in drafting the manuscript and revising it critically for important intellectual content. All authors read and approved the final manuscript.
